# Sodium Content and Labelling of Packaged Foods and Beverages in Nigeria: A Cross-Sectional Study

**DOI:** 10.3390/nu15010027

**Published:** 2022-12-21

**Authors:** Adedayo E. Ojo, Alexandra Jones, Clementina Ebere Okoro, Vanessa O. Alfa, Rosemary Okoli, Gabriel L. Shedul, Ikechukwu A. Orji, Samuel Osagie, Aashima Chopra, Linda V. Van Horn, Lisa R. Hirschhorn, Kathy Trieu, Fraser Taylor, Kylie Howes, Maliha Ilias, Makeda J. Williams, Veronica Tutse-Tonwe, Bruce Neal, Mark D. Huffman, Dike B. Ojji

**Affiliations:** 1 Cardiovascular Research Unit, University of Abuja and University of Abuja Teaching Hospital, Gwagwalada, Abuja 902101, Nigeria; 2 Department of Epidemiology and Global Health, University Medical Centre, Utrecht University, 3584 CG Utrecht, The Netherlands; 3 Department of Medicine, Faculty of Clinical Sciences, University of Abuja, Gwagwalada, Abuja 902101, Nigeria; 4 The George Institute for Global Health, University of New South Wales, Sydney, NSW 2052, Australia; 5 Department of Social Work, University of Nigeria, Nsukka 410105, Nigeria; 6 Department of Family Medicine, University of Abuja Teaching Hospital, Gwagwalada, Abuja 902101, Nigeria; 7 Department of Preventive Medicine, Feinberg School of Medicine, Northwestern University, Chicago, IL 60611, USA; 8 Department of Medical Social Sciences, Feinberg School of Medicine, Northwestern University, Chicago, IL 60611, USA; 9 National Heart, Lung, and Blood Institute, National Institutes of Health, Bethesda, MD 20814, USA; 10 School of Public Health, Imperial College London, London W2 1PG, UK; 11 Cardiovascular Division and Global Health Center, Washington University, St. Louis, MO 63110, USA

**Keywords:** sodium, salt, nutritional labelling, packaged foods, hypertension, cardiovascular diseases, Nigeria

## Abstract

Increased consumption of unhealthy processed foods, particularly those high in sodium, is a major risk factor for cardiovascular diseases. The nutrition information on packaged foods can help guide consumers toward products with less sodium and support government actions to improve the healthiness of the food supply. The aims of this study were to estimate the proportion of packaged foods displaying nutrition information for sodium and other nutrients specified by Nigerian nutrition labelling regulations and to determine the amount of sodium in packaged foods sold in Nigeria using data from the nutritional information panel. Data were collected from November 2020 to March 2021 from in-store surveys conducted in supermarkets in three states. A total of 7039 products were collected. Overall, 91.5% (*n* = 6439) provided only partial nutrition information, 7.0% (*n* = 495) provided no nutritional information, and only 1.5% (*n* = 105) displayed a nutrient declaration that included all nutrients specified by 2019 Nigerian regulations. Some form of sodium content information was displayed for 86% of all products (*n* = 6032), of which around 45% (*n* = 2689) expressed this as ‘salt’ and 59% (*n* = 3559) expressed this as ‘sodium’, while a small number of food products had both ‘salt’ and ‘sodium’ content (3.6%). Provision of sodium or salt information on the label varied between food categories, ranging from 50% (vitamins and supplements, *n* = 2/4) to 96% (convenience foods, *n* = 44/46). Food categories with the highest median sodium content were ‘meat and meat alternatives’ (904 mg/100 g), ‘sauces, dressings, spreads, and dips’ (560 mg/100 g), and ‘snack foods’ (536 mg/100 g), although wide variation was often observed within categories. These findings highlight considerable potential to improve the availability and consistency of nutrition information on packaged products in Nigeria and to introduce further policies to reduce the amount of sodium in the Nigerian food supply.

## 1. Background

Excess dietary intake of sodium increases the risk of high blood pressure, a major, modifiable risk factor for cardiovascular diseases (CVDs) [1,2]. CVDs and other diet-related non-communicable diseases (NCDs), including obesity, type 2 diabetes mellitus, and some cancers, are among the leading causes of global mortality in both high-income and low- and middle-income countries (LMIC) alike [3]. In Nigeria, the most populous country in Africa, high blood pressure is the leading cause of CVDs [4]. As in other LMIC contexts, Nigeria has experienced a nutrition transition over the past few decades, whereby traditional diets high in vegetables, fruits, and pulses have been replaced by an increasing proportion of highly processed and packaged foods [5]. The mean salt intake for Nigerian adults has been reported to be 7 g per day, which exceed the World Health Organization (WHO) daily recommendation of 2 grams (g) of sodium per day, which is equivalent to 5 g of salt per day [6,7]. The beneficial effects of reducing sodium intake on the prevention and control of high blood pressure are clear [8,9], yet the average global sodium intake is approximately 4 g per day or 10 g of salt per day [10]. Further, reducing sodium intake is a highly cost-effective strategy to control high blood pressure and prevent CVDs [11].

As part of the WHO Global Action Plan on the Prevention and Control of NCDs, WHO Member States have endorsed a global target of a 30% relative reduction in mean population intake of sodium, intending to achieve a target of less than 2 g of sodium (i.e., <5 g of salt) per day by 2025 [12]. Among its ‘best-buy’ strategies for highly cost-effective and feasible approaches to reducing population sodium intake, the WHO includes nutrition labelling reforms including back-of-pack nutrition information to quantify sodium content, and front-of-pack nutrition labelling to improve understanding and use of this information. Other ‘best-buy’ strategies include sodium reformulation targets for processed and packaged foods, provision of low sodium options in public settings such as schools and hospitals, and behaviour change through mass media campaigns [13]. In the context of sodium reduction specifically, WHO has synthesised these strategies through the SHAKE package (Surveillance of salt intake, Harness industry, Adopt standards for labelling and marketing, Knowledge to empower consumers, and Environments to promote healthy eating) to support a comprehensive approach for governments to maximise public health impact.

In 2019, Nigeria announced its National Multi-sectoral Action Plan (NMSAP) for NCDs, which included a target to reduce the mean population dietary sodium intake by at least 30% by 2025 in alignment with the WHO global target [14,15]. Meeting this target will require implementing the relevant WHO best buys and associated SHAKE strategies. In the area of nutrition labelling, Nigerian regulations, introduced in 2019, only require mandatory nutrient declarations on the back of pre-packaged foods and beverages that make a health or nutrient claim [16]. Where nutrient declarations are provided, the regulation specifies that this information can be provided per 100 g, per 100 mL, or per serving but should contain information on energy value, amounts of fat (specifying saturated and trans fat), carbohydrate (specifying the quantity from total sugars), protein, salt, and the amount of any other nutrient for which a nutrition or health claim is made [16]. While elements of these updated regulations are promising in that salt is now included as a required nutrient for the first time, the law still falls short of the international Codex Alimentarius Commissions Guidelines on Nutrition Labelling, [17], which recommend nutrient declarations be mandatory on *all* pre-packaged foods. The Codex Alimentarius also suggest nutrient declarations generally be presented on a standardised per 100 g or 100 mL basis to facilitate comparison and avoid confusion if based on serving size. This differs from Nigeria’s current allowance for manufacturers to provide nutrient declarations on a per serving basis.

To improve upon back-of-pack nutrition information, governments worldwide are following WHO recommendations to implement front-of-pack nutrition labelling (FOPNL) on packaged foods and beverages [8]. These simple, graphical labels use words and colours to provide at-a-glance information on nutritional quality to complement back-of-pack nutrient declarations. Existing authoritative guidance from both WHO and Codex support government action in this area [18], but provide limited detail on the appropriate content of national FOPNL regulation. In Nigeria, there have been efforts led by the Nigerian Heart Foundation to initiate a voluntary ‘Heart Tick’ style label on pre-packaged foods to identify heart-healthy foods [8], but there has yet to be any government-led FOPNL scheme developed or implemented in Nigeria.

Beyond these limited activities related to nutrition labelling, Nigeria has not yet commenced major policy activities in WHO’s other recommended areas of action on sodium. To provide a baseline estimate to guide national policy action, the objective of this study was to estimate the proportion of packaged foods and beverages displaying nutrition information for sodium and other nutrients specified by Nigerian nutrition labelling regulations and to determine the amount of sodium in packaged foods sold in Nigeria.

## 2. Methods

This study was a cross-sectional survey of all available commercially packaged foods and beverages in leading Nigerian retail stores in capital cities of the Federal Capital Territory, Kano, and Ogun states in Nigeria between November 2020 and March 2021. Using similar methods from previous studies [19,20], large and frequently visited retail supermarkets and stores were purposively chosen for data collection because these stores sell products that are widely sold and consumed. This retail survey is part of the larger, multi-faceted Nigeria Sodium Study, which seeks to evaluate Nigeria’s implementation of its dietary sodium policies, as detailed elsewhere [21].

### 2.1. Data Source

**Figure 1:**
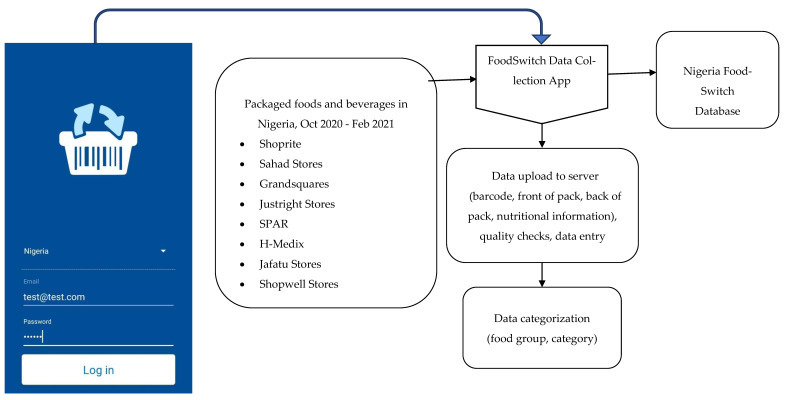
Overview of the development of the Nigeria FoodSwitch Database, 2020.

The George Institute for Global Health created its FoodSwitch system to collect information about the food supply in multiple jurisdictions worldwide. To support this study, we gained permission from store managers to collect data from leading retailers in three Nigerian states. The FoodSwitch Data Collector App was used by trained data collection officers who used study smartphones to scan the barcode and photograph the front- and back-of-food packaging, capturing information such as product names, nutrient declarations, ingredient lists, and manufacturer information. Photographs were uploaded into a cloud-based server, and then data were stored, extracted, and categorised using a standardised method by trained research staff [22] (Figure 1).

### 2.2. Food Labelling and Food Composition Data

During data collection, we captured the presence or absence of a partial or complete nutrient declaration on pack. Where a nutrient declaration was present, we extracted available information on the following nutrients, where necessary converting information into the following standardised measurements: energy (kJ/100 g), protein (g/100 g), saturated fat (g/100 g), trans fat (g/100 g), carbohydrate (g/100 g), total sugar (g/100 g), sodium (mg/100 g), and salt (g/100 g). We defined a partial nutrient declaration as including information on at least one of the components specified in the Nigerian regulation, and a complete nutrient declaration as providing information on *all* components specified in the 2019 Nigerian regulation, i.e., energy, saturated fats, trans fats, carbohydrates, total sugars, protein, and either sodium or salt. We also extracted information on product manufacturers.

### 2.3. Data Categorisation

Products were grouped and categorised using the system developed by the Global Food Monitoring Group (GFMG) [19,20,21,22,23], which was based on previous reports, existing branded food databases, and expert consensus to optimise international comparison and interpretation by industry, and other stakeholders. This hierarchical system is designed to monitor the nutrient composition of food products globally [24]. Products were classified by trained dietitians and nutritionists into 16 major food categories: (a) bread and bakery products, (b) cereal and grain products, (c) confectionery, (d) convenience foods, (e) dairy, (f) edible oils and oil emulsions, (g) alcohol, (h) foods for specific dietary use, (i) fruits, vegetables, nuts and legumes, (j) meat and meat alternatives, (k) non-alcoholic beverages, (l) sauces, dressings, spreads, and dips, (m) seafood and seafood products, (n) snack foods, (o) sugars, honey and related products, and (p) vitamins and supplements. Products were identified by their unique barcode.

### 2.4. Statistical Analyses

The number and percentage of products displaying partial and complete nutrient declarations were reported, along with the number and percentage of products that displayed sodium information expressed either as sodium or salt, overall and by category. Salt per 100 g was converted to sodium per 100 mg by multiplying salt figure by 400. Descriptive statistics (i.e., medians, interquartile ranges, and ranges of sodium levels per 100 g) were also calculated and presented for each sub-category. All statistical analyses were conducted using SAS version 9.4 (The SAS Institute, Cary, NC, USA).

## 3. Results

Data were collected from retail outlets in Federal Capital Territory (*n* = 16), Kano (*n* = 17), and Ogun (*n* = 12) states.

### 3.1. Labelling Completeness of Packaged Food Products in Nigeria

A total of 7039 packaged foods and beverages made by 1089 unique manufacturers across 16 major food groups and 89 food categories were identified in the Nigerian supermarkets surveyed. The major food groups with the largest number of products were ‘bread and bakery products’ (*n* = 1352, 19.2%), followed by ‘fruits, vegetables, nuts, and legumes’ (*n* = 1015, 14.4%) and ‘non-alcoholic beverages’ (*n* = 889, 12.6%) (Table 1).

We identified 6439 (91.5%) products with a partial or incomplete nutrient declaration; however, only 105 products (1.5%) displayed complete nutrient declaration in compliance with the 2019 update to Nigeria’s nutrition labelling regulation. The remaining 495 (7.0%) products displayed no nutrient information (Table 2). The information most frequently missing from incomplete nutrient declarations was trans fats (only present on 26% of products) and sodium expressed as ‘salt’ as per the requirements of regulation (only present on 38% of all products) (Supplementary Figure S1).

Information on sodium content was obtained from the labels of 6032 (85.7%) products. Of these, 59.0% (*n* = 3559) provided this information expressed as sodium, and 44.6% (*n* = 2689) displayed this information as ‘salt’. A small number of products (*n* = 216; 3.6%) provided information as both sodium and salt (Supplementary Table S1). The form of presenting this information in the nutrient declaration also varied, with 61.4% (*n* = 3704) products providing information on both a standardised per 100 g/mL basis and per serving, 37.1% (*n* = 2238) presenting information based on per 100 g only, and 1.5% (*n* = 90) presenting information on a per serving basis only (Supplementary Figure S2).

**Figure 2:**
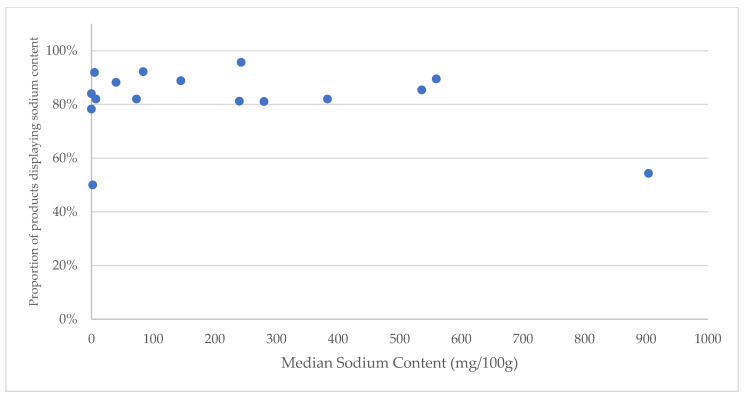
Prevalence of sodium labelling and median sodium content reported for the 16 defined categories of packaged foods and beverages in Nigerian retail stores by median sodium context, 2020–2021.

Provision of sodium information varied by category and was most prevalent in the categories of ‘convenience foods’ (*n* = 42/44, 96%), ‘confectionary’ (*n* = 566/615, 92%), ‘sugars, honey, and related products’ (*n* = 135/147, 92%), and ‘sauces, dressings, spreads, and dips’ (*n* = 484/544, 89%). Provision of sodium information was lowest in the categories of ‘meat and meat alternatives’ (*n* = 24/44, 55%) and ‘vitamins and supplements’ (*n* = 2/4, 50%) (Figure 2).

Provision of sodium information also varied by manufacturer. Of the 1089 unique manufacturers, 430 (39.5%) displayed sodium or salt information on all products, 209 (19.2%) had sodium or salt information on some products, and 449 (41.2%) did not present sodium or salt information at all (Supplementary Table S2).

### 3.2. Sodium Content of Packaged Foods Available in Nigeria

The declared sodium content was highly variable between food groups and categories (Figure 2). The major food groups with the highest median sodium content were ‘meat and meat alternatives’ (904 mg/100 g), ‘sauces, dressings, spreads, and dips’ (559.5 mg/100 g) and ‘snack foods’ (536 mg/100 g). Major food groups with the lowest median sodium content (mg/100 g) were ‘vitamins and supplements (2 mg/100 g), ‘sugars, honey, and related products’ (5 mg/100 g) and ‘non-alcoholic beverages’ (7.2 mg/100 g)**.**

For some high sodium food groups, information on sodium content was more limited; for example, sodium labelling was only present on 54% (*n* = 44) of ‘meat and meat alternatives’ despite this being the group with the highest median content. Similarly, products such as ‘edible oils and oil emulsion’ and alcohol displayed labels with zero sodium content among 84% (*n* = 179) and 78% (*n* = 36) of products, respectively. Packaged foods and beverages with zero sodium content had about 50% sodium labeling, and this was similar to products reported with about 900 mg/100g; whereas, products reported with sodium content between 200 mg/100g and 300 mg/100g were reported to have highest sodium labeling (Figure 2).

## 4. Discussion

In this study, we assessed the prevalence and completeness of the nutrient declaration, the prevalence of sodium labelling on the pack, and the reported sodium content of food and beverage products available in Nigerian retail markets in three major cities. This is the largest retail survey of its kind in Nigeria to date, and so while the sampling frame may not have been representative, it is likely to have captured the most commonly sold and consumed packaged foods and beverages. Overall, 91.5% (*n* = 6439) provided only partial nutrition information, 7.0% (*n* = 495) provided no nutritional information, and only 1.5% (*n* = 105) displayed a nutrient declaration that included all nutrients specified by 2019 Nigerian regulations. Sodium content of packaged foods and beverage were found to be highest for ‘meat and meat alternatives’, ‘sauces, dressings, spreads, and dips’, and ‘snack foods’.

Our investigation revealed that very limited packaged foods and beverages provided complete nutritional information with reference to the 2019 Nigeria food labelling regulations. Food products and beverages were more likely to have partial nutritional information available on the pack of the products. The 2019 Nigeria food labelling regulations required that salt should be displayed on the product package, but less than half of the packaged food products and beverages met this regulation. The overall sodium labelling of packaged foods and beverages in Nigeria (sodium = 45%; salt 59%) was found to be higher compared to what was previously reported in a study conducted in Kenya, where 39% of packaged food products displayed sodium content; this was also the same for another study conducted in India, where only 32% sodium labelling was reported. Sodium content of packaged foods and beverage were found to be highest for ‘meat and meat alternatives’, ‘sauces, dressings, spreads, and dips’, and ‘snack foods’.

Provision of consistent and understandable nutrient information through accurate and complete back-of-pack nutrient declarations is important to inform consumer choice and promote transparency in the food supply. Our results show that very few products in Nigeria displayed nutrient declarations that fully comply with the information required by the 2019 nutrition labelling regulations, though the vast majority of products provided at least partial information. This may be in part related to the relatively recent introduction of new regulations, and also related to those regulations only requiring nutrient declarations to be provided on a mandatory basis on products which make a health or nutrient claim, but also suggests significant scope to improve uptake.

Other elements of our analysis of nutrition labelling information also suggest areas for improving the utility of Nigeria’s food labels for consumers. Among Nigerian products currently displaying a partial or complete nutrient declaration, inconsistencies in the way this information is provided, for example as both salt and sodium, and not consistently using a per 100 g or 100 mL standardised presentation, also limit the usability of this information to consumers. At a minimum, Nigeria could strengthen front-of-pack nutrition labelling requirements to be consistent with international *Codex Alimentarius* which require mandatory nutrient declarations for all pre-packaged foods and suggest that nutrient declarations be generally presented using a standardised per 100 g or 100 mL basis to facilitate comparisons between foods.

Improved back-of-pack nutrient declarations are also necessary to support a variety of other WHO ‘best-buy’ policies to address unhealthy diets and prevent and control NCDs. These policies include reformulation programs and targets, procurement standards for public settings such as schools and hospitals, and front-of-pack nutrition labelling systems, each of which relies on information drawn from accurate nutrient declarations for effective implementation [25,26]. For Nigeria to make progress toward its National Multi-Sectoral Action Plan target to reduce the mean population′s dietary sodium intake by at least 30% by 2025, further action should be considered to improve nutrition labelling to facilitate the implementation of these policies. Nigeria can learn from other countries that have developed sodium reduction policies, such as the UK that has successful implemented a reformulation program on salt reduction strategies, and nearby South Africa which has implemented mandatory sodium limits in regulation for certain food categories [27].

Our study findings on reported sodium content are consistent with previous studies which have identified the food groups that have the highest levels and concentrations of sodium content in the food supply [28]. For example, in South Africa, snack foods, meat and meat alternatives, and sauces and spreads were similarly reported to be food groups with the highest median sodium content. Similarly, confectionary foods and dairy were both reported in Nigeria and South Africa as the food groups with the lowest sodium content. Variability in sodium content within categories of foods highlights the potential for reformulation programs to reduce the sodium content [29].

To reduce the burden of CVDs, sodium reduction is one of the major priority actions globally [30,31]. In Nigeria, there is an urgent need for the adoption of a sodium reduction strategy incorporating a range of WHO best-buy policies. Without stronger action to improve the food environment, there is unlikely to be meaningful progress towards reducing population sodium intake.

The strength of this study is the fact that it included a large number of packaged products in the Nigerian food and beverage supply with data captured directly from retail stores using a structured and widely reported approach. An important limitation of the current study is that data were collected from supermarkets and stores from three cities; thus, these results may not be nationally representative of all food and beverage products sold in Nigeria but likely provide the best estimate of commonly available packaged foods and beverages. Future research from this study will collect and evaluate information on unpackaged and street/restaurant foods and beverages. Another limitation in this study was that we relied on the validity and accuracy of the nutritional information displayed on the NIP of the food products. While it may be possible the chemical analysis of food products may provide more precision information about the nutritional content of packaged foods and beverages, this approach is expensive and was outside the scope of the current study. Finally, the lack of sales data to understand the potential impact of findings was also a challenge.

## 5. Conclusions

These findings highlight considerable potential to improve the availability and consistency of nutrition information on packaged products in Nigeria and to introduce further actions to reduce the amount of sodium in the Nigerian food supply. Low nutrient declaration and sodium labelling of packaged foods and beverages call for interventions of the relevant stakeholders in Nigeria. To improve the utility of food labels for consumers and improve transparency in the food supply, further action could be considered by the Nigerian stakeholders to strengthen nutrition labelling by requiring complete nutrient declarations for all packaged products. Taking this step will also facilitate the implementation of other recommended policies for sodium reduction, including reformulation programs and front-of-pack nutrition labelling to work towards Nigeria’s targeted 30% reduction in population sodium intake by 2025.

## Figures and Tables

**Table 1:** Overview of sodium labelling and content of packaged foods by major food group, and food categories in Nigeria, 2021.

Food Group	Food Category	Total Number of Products	Products Displaying Sodium Content Information (%)	Sodium Content		
				Median (mg/100 g)	IQR (mg/100 g)	Range (mg/100 g)
Bread and bakery products	Biscuits/cookies/crackers	1240	80.9	273.0	186	0–40,000
	Bread	20	90.0	568.5	1114.1	274.8–4000
	Cakes, muffins, and pastries	92	82.6	385.5	861.0	30–1533
Cereal and grain products	Breakfast cereals	373	90.6	198.0	387.0	0–6800
	Couscous	8	75.0	9.0	6.0	0–38
	Miscellaneous cereal and grain products	183	78.7	35.0	400.0	0–38,500
	Noodles	59	91.5	760.0	1342.0	0–1900
	Pasta	143	92.3	4.0	7.8	0–1029
	Rice and rice products	74	90.5	2.0	12.0	0–2000
Confectionery	Chewing gum	46	95.7	8.0	16.0	0–505.8
	Chocolate and sweets/candy	592	91.7	96.0	116.0	0–31,000
	Cough lozenges	1	100.0	21.5	0.0	21.5–21.5
	Jelly (Jell-O)	28	96.4	12.0	12.0	0–120
Convenience foods	Frozen Asian dumplings and similar products	3	66.7	509.0	262.0	378–640
	Pre-prepared salads and sandwiches	4	100.0	360.0	480.0	320–1200
	Ready meals	12	91.7	320.0	170.0	40–512
	Soup	27	100.0	236.0	68.0	21–5600
Seafood and seafood products	Canned seafood	117	83.8	383.0	60.0	80–6360
	Dried seafood	12	58.3	400.0	408.0	11–784
	Fish spread	2	100.0	400.0	160.0	320–480
	Frozen seafood	4	100.0	488.5	507.5	360–1160
	Seafood and seafood products not otherwise specified	4	75.0	1107.0	980.0	180–1160
Dairy	Cheese	49	91.8	776.0	360.0	14–1800
	Cream	19	94.7	32.0	30.0	0–248
	Desserts	59	81.4	71.1	178.0	4–762
	Ice cream/edible ices	21	90.5	100.0	108.0	12–241
	Milk	224	78.6	56.0	188.0	0–1221
	Probiotic drinks	2	100.0	8.0	0.0	8–8
	Yoghurt/yoghurt drinks	38	78.9	36.0	16.0	8–113
Foods for specific dietary use	Baby and infant foods	146	90.4	146.0	167.0	0–367
	Diet drink mixes (meal replacements)	13	100.0	89.0	162.0	0.4–360
	Foods for specific dietary use not otherwise specified	16	75.0	1.8	270.0	0–2280
	Sports products	4	50.0	124.0	212.0	18–230
Fruits, vegetables, nuts, and legumes	Fruit and fruit products	91	95.6	5.9	40.0	0–5533
	Herbs and spices	136	60.3	200.0	4983.0	0–35,833
	Jam (jelly in the US) and marmalades	64	81.3	8.0	40.0	0–195
	MSG	1	100.0	12,000.0	0.0	12,000–12,000
	Nuts and seeds	154	87.0	166.0	412.0	0–11,000
	Salt, plain and flavoured	21	47.6	29,125.0	26,256.0	0–39,680
	Seasoning	183	65.0	21,910.0	14,500.0	0–37,500
	Vegetables	365	92.9	268.0	565.0	0–24,000
Non-alcoholic beverages	Beverage mixes	154	79.9	133.0	279.0	0–10,500
	Breakfast beverages	24	100.0	145.0	160.0	46–710
	Coffee and tea	284	87.7	0.0	107.0	0–37,000
	Cordials (syrups)	7	100.0	0.0	0.0	0–24
	Electrolyte (sports) drinks	7	100.0	0.0	0.0	0–53
	Energy drinks	48	91.7	40.0	51.5	0–125
	Fermented beverages (e.g., Kombucha)	2	50.0	66.0	0.0	66–66
	Fruit and vegetable juices	218	78.9	4.0	10.0	0–9100
	Non-alcoholic beverages not otherwise specified	9	77.8	10.0	383.0	0–2000
	Soft drinks	111	74.8	4.0	8.2	0–21,000
	Waters	25	48.0	16.0	18.0	0–600
Sauces, dressings, spreads and dips	Mayonnaise/salad dressings	153	98.0	535.0	385.0	0–1867
	Sauces	367	84.2	680.0	974.0	0–22,000
	Spreads and dips	88	96.6	391.0	448.0	0–1640
Alcohol	Alcohol not otherwise specified	1	100.0	0.0	0.0	0–0
	Beer	3	33.3	0.0	0.0	0–0
	Cordial, mixers and syrups (integrated alcoholic beverages)	3	66.7	41.5	77.0	3–80
	Liquor (distilled spirits)	11	54.5	21.5	37.0	0–60
	Wine	28	92.9	0.0	4	0–18,800
Edible oils and oil emulsions	Cooking oils	155	86.5	0.0	0.0	0–11,100
	Edible oils	58	77.6	470.0	981.0	0–1870
Meat and meat alternatives	Meat alternatives	2	100.0	0.0	0.0	0–0
	Processed meat	79	53.2	912.0	460.0	85.1–7360
Sugars, honey and related products	Condensed caramel	1	100.0	60.0	0.0	60–60
	Dessert additions (e.g., sprinkles)	1	100.0	48.0	0.0	48–48
	Dessert toppings (e.g., caramel sauce)	5	100.0	163.0	175.0	4–200
	Food essence and colouring	13	92.3	0.0	300.0	0–6360
	Honey and pollen	37	94.6	3.0	12.0	0–40
	Icing	6	83.3	0.0	4.0	0–258
	Sugar	16	87.5	5.2	15.0	0–120
	Sugars, honey, and related products not otherwise specified	6	100.0	30.5	80.0	0–240
	Sweeteners	27	77.8	0.0	36.0	0–9120
	Syrups	48	97.9	23.0	166.1	0–333
Snack foods	Cassava chips	6	100.0	394.0	331.0	308–679
	Cereal/nut-based snack bars	35	94.3	228.0	250.4	12–756.4
	Corn-based snacks/chips	20	75.0	476.0	290.8	76.1–1067
	Extruded snacks	1	100.0	1200.0	0.0	1200–1200
	Legume-based snacks/chips (e.g., Wasabi peas)	9	77.8	413.6	633.0	0–1040
	Noodle-based snacks/chips	3	100.0	401.0	621.0	357–978
	Popcorn	37	64.9	490.5	698.5	3.6–1188
	Potato-based snacks and chips/crisps	137	92.7	640.0	326.0	40–1887
	Pretzels	2	100.0	850.0	100.0	800–900
	Rice-based snacks	12	91.7	113.0	485.0	47.5–1182
	Salt and vinegar flavoured snacks (excl. Potato chips)	7	85.7	762.5	719.0	40–2000
	Snack foods not otherwise specified	76	77.6	474.0	642.0	0–1900
	Snack packs	21	85.7	736.0	285.0	0–1087
	Vegetable-based snacks/chips	9	88.9	519.5	342.5	140–1040
	Wholegrain chips	2	100.0	930.0	340.0	760–1100
Vitamins and supplements	–	4	50.0	2.0	4.0	0–4

11 products were excluded from the table as they were unable to be categorised.

**Table 2:** Nutrition labelling of foods and beverages in the Nigerian food supply.

	Total Number of Products (N)	Percent of Food Supply (%)
Complete nutrient declaration: provides a nutrient declaration with information on all food components required by 2019 regulation	105	1.5
Partial nutrient declaration: provides a nutrient declaration with at least one nutrient from energy, saturated fat, trans fat, carbohydrates, sugars, protein, salt.	6439	91.5
No nutrient declaration	495	7.0
	7039	

MSG: Monosodium glutamate.

## Data Availability

Restricted data were accessed remotely through the FoodSwitch database.

## Supplementary Materials

The following are available online at https://www.mdpi.com/article/10.3390/nu15010027/s1, Table S1: Provision of sodium and/or salt information on packaged foods in Nigeria. Table S2: Distribution of packaged foods products in Nigeria by the compliance of sodium labelling by manufacturers. Figure S1: Presentation of information on specific nutrients required by Nigerian regulation within the nutrient declaration of foods and beverages in the Nigerian food supply. Figure S2: Sodium/Salt content of foods and beverages in the Nigerian food supply, 2020–2021.
