# Supplementary material for: Sodium Content and Labelling of Packaged Foods and Beverages in Nigeria: A Cross-Sectional Study
Source: Nutrients. 2022 Dec 21;15(1):27. doi: 10.3390/nu15010027 (PMC9823880; doi:10.3390/nu15010027)
Supplement: Supplementary file 1 [file nutrients-15-00027-s001.zip › nutrients-2050165-supplementary.pdf]

Supplementary Materials

Table S1. Provision of sodium and/or salt information on packaged foods in Nigeria.

	**Number of products displaying information, n**	**Proportion of products Displaying information, %**
Sodium	3559	50.1%
Salt	2689	38.2%
Overall Sodium or Salt	6032	85.7%

*Note that 216 products contained both sodium and salt information

Table S2. Distribution of packaged foods products in Nigeria by the compliance of sodium labeling by manufacturers.

Total manufacturers (N=1,089)	Total Products (N=7,039)
Manufacturers with 100% of products with sodium labeling, n=430 (39.5%)	1,266 (18%)
Manufacturers with 1-99% of products with sodium labeling, n=209 (19.2%)	2,695 (38%)
Manufacturers with 0% of products with sodium labeling, n=449 (41.2%)	1,423 (20%)
Unknown Manufacturer	1,655 (24%)

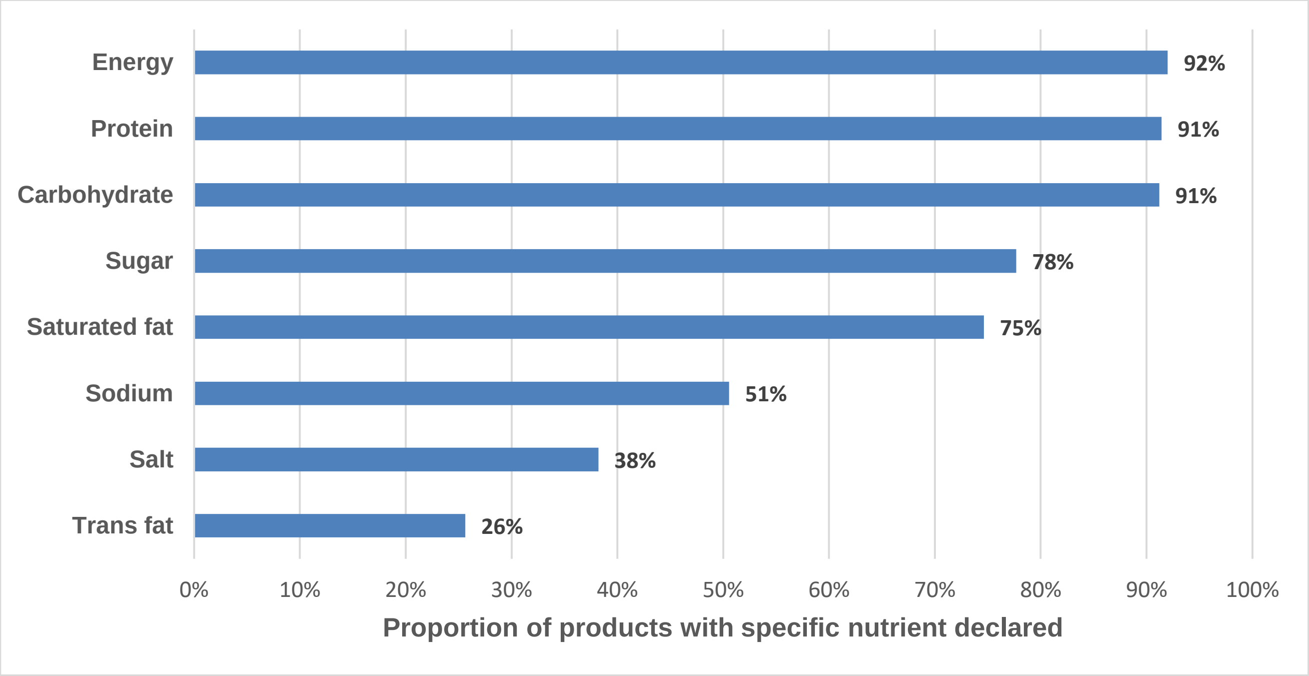

N.B. Under the 2019 Nigerian Food Labelling Regulation, sodium information is to be provided as salt in the nutrient declaration. This explains the division of this information over two bars.

Figure S1. Presentation of information on specific nutrients required by Nigerian regulation within the nutrient declaration of foods and beverages in the Nigerian food supply.

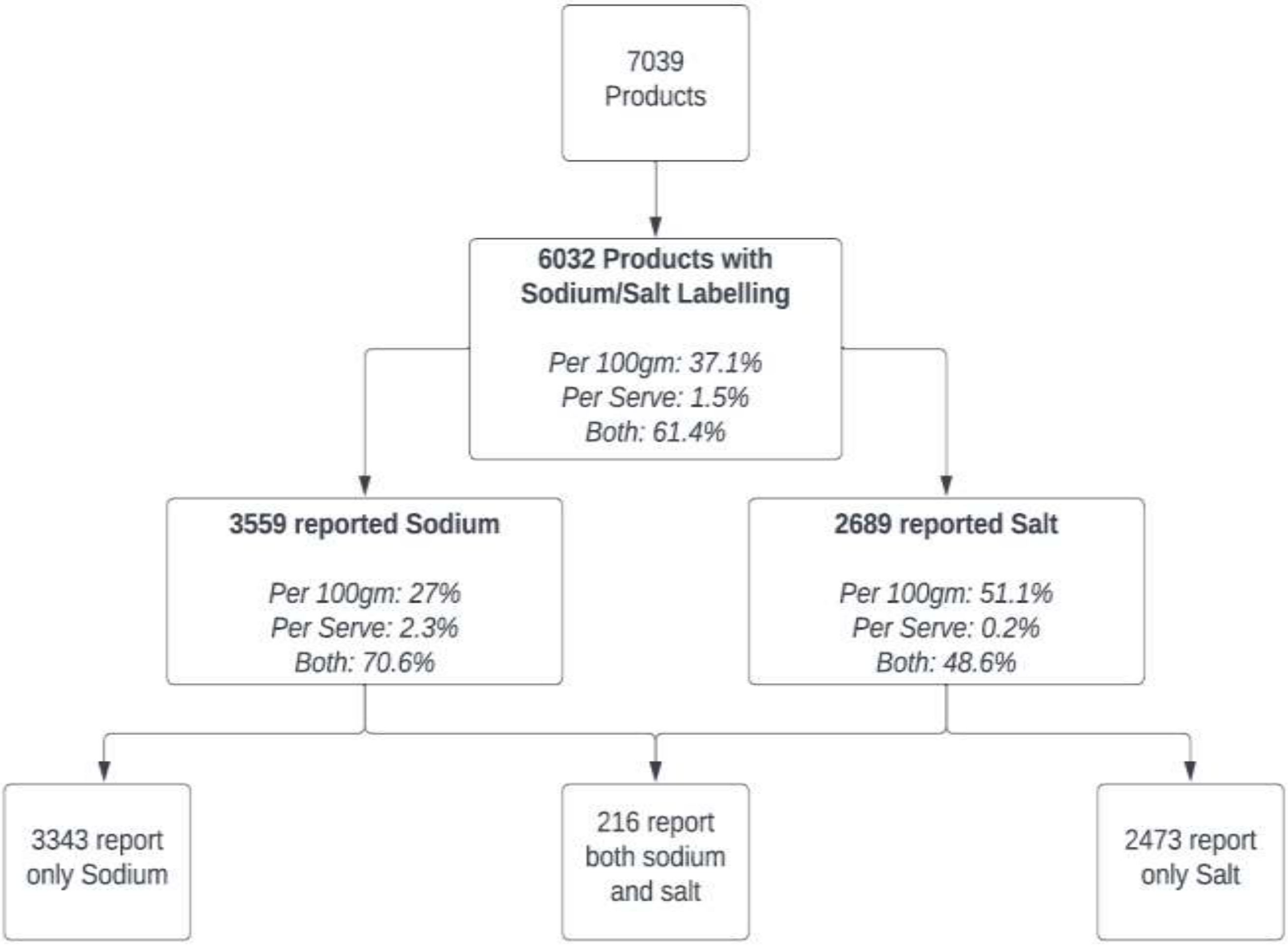

Figure S2. Sodium/Salt content of foods and beverages in the Nigerian food supply, 2020-2021
